# Epidemiology and Burden of Ventilator-Associated Pneumonia among Adult Intensive Care Unit Patients: A Portuguese, Multicenter, Retrospective Study (eVAP-PT Study)

**DOI:** 10.3390/antibiotics13040290

**Published:** 2024-03-22

**Authors:** Paulo Mergulhão, João Gonçalves Pereira, Antero Vale Fernandes, Andriy Krystopchuk, João Miguel Ribeiro, Daniel Miranda, Heloísa Castro, Carla Eira, Juvenal Morais, Cristina Lameirão, Sara Gomes, Dina Leal, Joana Duarte, Leonor Pássaro, Filipe Froes, Ignacio Martin-Loeches

**Affiliations:** 1 Intensive Care Unit, Hospital Lusíadas Porto, 4050-115 Porto, Portugal; paulo.mergulhao.gomes@lusiadas.pt; 2 Intensive Care Unit, Hospital de Vila Franca de Xira, 2600-009 Vila Franca de Xira, Portugal; joao.goncalvespereira@hvfx.min-saude.pt; 3 Intensive Care Unit, Hospital Garcia de Orta, 2805-267 Almada, Portugal; antero.fernandes@hgo.min-saude.pt; 4 Intensive Care Unit, Centro Hospitalar Universitário do Algarve, 8000-386 Faro, Portugal; akrystopchuk@chalgarve.min-saude.pt; 5 Intensive Care Unit, Hospital de Santa Maria, Centro Hospitalar Universitário Lisboa Norte, 1649-035 Lisbon, Portugal; jmribeiro@ulssm.min-saude.pt; 6 Intensive Care Unit, Centro Hospitalar Vila Nova de Gaia e Espinho, 4434-502 Vila Nova de Gaia, Portugal; daniel.miranda@chvng.min-saude.pt; 7 Intensive Care Unit, Hospital de Santo António, Centro Hospitalar Universitário do Porto, 4099-001 Porto, Portugal; u08484@chporto.min-saude.pt; 8 Intensive Care Unit, Centro Hospitalar Tondela Viseu, 3504-509 Viseu, Portugal; carla.eira.6452@hstviseu.min-saude.pt; 9 Intensive Care Unit, Hospital São Francisco Xavier, Centro Hospitalar Lisboa Ocidental, 1449-005 Lisbon, Portugal; juvenal.jf.morais@azores.gov.pt; 10 Intensive Care Unit, Centro Hospitalar Trás-os-Montes e Alto Douro, 5000-508 Vila Real, Portugal; cgomes@chtmad.min-saude.pt; 11 Intensive Care Unit, Hospital Prof. Doutor Fernando Fonseca, 2720-276 Amadora, Portugal; sara.r.gomes@hff.min-saude.pt; 12 Intensive Care Unit, Hospital de Braga, 4710-243 Braga, Portugal; dina.leal@hb.min-saude.pt; 13 Medical Affairs Department, MSD Portugal, 2770-192 Oeiras, Portugal; joana.duarte@merck.com (J.D.); leonor.passaro@ulsetejo.min-saude.pt (L.P.); 14 Intensive Care Unit, Hospital Pulido Valente, Centro Hospitalar Universitário Lisboa Norte, 1769-001 Lisbon, Portugal; filipe.froes@ulssm.min-saude.pt; 15 Department of Intensive Care Medicine, Multidisciplinary Intensive Care Research Organization (MICRO), St James’ Hospital, D08NYH1 Dublin, Ireland

**Keywords:** hospital stay, invasive mechanical ventilation, nosocomial infections, sepsis, VAP (ventilator-associated pneumonia)

## Abstract

Ventilator-associated pneumonia (VAP) is a prevailing nosocomial infection in critically ill patients requiring invasive mechanical ventilation (iMV). The impact of VAP is profound, adversely affecting patient outcomes and placing a significant burden on healthcare resources. This study assessed for the first time the contemporary VAP epidemiology in Portugal and its burden on the healthcare system and clinical outcomes. Additionally, resource consumption (duration of iMV, intensive care unit (ICU), hospital length of stay (LOS)) and empirical antimicrobial therapy were also evaluated. This multicenter, retrospective study included patients admitted to the hospital between July 2016 and December 2017 in a participating ICU, who underwent iMV for at least 48 h. Patients with a VAP diagnosis were segregated for further analysis (*n* = 197). Control patients, ventilated for >48 h but without a VAP diagnosis, were also included in a 1:1 ratio. Cumulative VAP incidence was computed. All-cause mortality was assessed at 28, 90, and 365 days after ICU admission. Cumulative VAP incidence was 9.2% (95% CI 8.0–10.5). The all-cause mortality rate in VAP patients was 24.9%, 34.0%, and 40.6%, respectively, and these values were similar to those observed in patients without VAP diagnosis. Further, patients with VAP had significantly longer ICU (27.5 vs. 11.0 days, *p* < 0.001) and hospital LOS (61 vs. 35.9 days, *p* < 0.001), more time under iMV (20.7 vs. 8.0 days, *p* < 0.001) and were more often subjected to tracheostomy (36.5 vs. 14.2%; *p* < 0.001). Patients with VAP who received inappropriate empirical antimicrobials had higher 28-day mortality, 34.3% vs. 19.5% (odds ratio 2.16, 95% CI 1.10–4.23), although the same was not independently associated with 1-year all-cause mortality (*p* = 0.107). This study described the VAP impact and burden on the Portuguese healthcare system, with approximately 9% of patients undergoing iMV for >48 h developing VAP, leading to increased resource consumption (longer ICU and hospital LOS). An unexpectedly high incidence of inappropriate, empirical antimicrobial therapy was also noted, being positively associated with a higher mortality risk of these patients. Knowledge of the Portuguese epidemiology characterization of VAP and its multidimensional impact is essential for efficient treatment and optimized long-term health outcomes of these patients.

## 1. Introduction

Ventilator-associated pneumonia (VAP) is a nosocomial infection that occurs in patients undergoing invasive mechanical ventilation (iMV) for more than 48 h [1] and ranks among the most prevalent nosocomial infections in intensive care units (ICU) [2]. Several epidemiological studies have demonstrated substantial variations in VAP incidence across countries and ICU types. The 2017 European Centre for Disease Prevention and Control patient-based surveillance of ICU-acquired infections disclosed that 97.3% of 8983 pneumonia cases in ICUs were linked to intubation [3]. A median of 9.5 intubation-associated pneumonia episodes per 1000 intubation days was noted, ranging between 2.1 (Luxembourg) and 13.7 (Hungary) [3]. In Portugal, the same authors reported an average prevalence of 7.2 VAP cases per 1000 intubation days per year [3]. Notably, higher VAP rates have been observed in cancer (24.5%) [4] and trauma patients (17.8%) [5], which suggests that VAP prevalence is much dependent on the ICU case mix. Additionally, VAP can be further categorized into early and late onset, with early-onset VAP occurring within 96 h of MV, and late-onset VAP occurring after 96 h of initiation of MV, with the latter being usually caused by multidrug-resistant (MDR) pathogens, leading to increased morbidity and mortality [6,7]. Although VAP-attributable mortality can be significant [8], it appears to be declining over time [9]. Findings from a comprehensive systematic review focusing on the occurrence of VAP in intensive care units across low-, middle-, and high-income countries revealed a noteworthy variation in VAP occurrence rates, spanning from 6.3% to 66.9%. This comprehensive analysis also underscored a considerable heterogeneity observed in the mortality rates attributed to VAP [10].

The use of iMV is highly associated with increased morbidity, mortality, and healthcare costs [8], and the association between iMV and VAP is well established [11]. Tracheal intubation and iMV circumvent the defense mechanisms of the respiratory system and interfere with its normal protection reflexes, hindering effective coughing and mucociliary clearance and promoting the microaspiration of contaminated oropharyngeal contents [12]. Most cases of VAP are caused by the microaspiration of oropharyngeal secretions that accumulate above the cuff of the endotracheal or tracheostomy tube and by the aspiration of bacterial biofilm that forms on the inner surface of the endotracheal tube [13]. Also, mechanical ventilation duration is associated with the risk of VAP, with a cumulative risk of 3% per day during the first week, and 2% per day on the second week of ventilation. The highest risk of infection occurs between 8 and 10 days [14]. It has been estimated that approximately 50% of antibiotics administered in the ICU are used for the management of VAP cases [15].

There are various risk factors for VAP onset. Male sex, underlying disease severity, and a history of trauma are various independent risk factors that lead to the development of VAP [16]. Chronic conditions have also been documented as risk factors for VAP, including coronary heart disease, diabetes, and chronic renal failure [16]. However, current VAP diagnosis still relies on unspecific clinical, radiographic signs and microbiological criteria [1].

Microbiological assessment is important to document infection, enabling tailored therapy adjustments [17]. Studies on VAP epidemiology and microbiological causative agents documented that *P. aeruginosa*, *S. aureus*, and *K. pneumoniae* are the most prevalent agents to cause VAP in intensive care units [3,18]. In Portugal, *P. aeruginosa* (27.4%) and *K. pneumoniae* (25.4%) were the two main microorganisms isolated from ICU patients with pneumonia in 2019 [19]. Positive correlations were also found between antibiotic consumption in ICU patients and the potential isolation of multidrug-resistant bacteria, especially on *P. aeruginosa* and *S. aureus* [20].

VAP is associated with difficulty in weaning from the ventilator and with longer ICU and hospital length of stay (LOS), which causes an enormous financial burden to patients and facilities, and a huge demand for medical resources [13]. Accordingly, patients with VAP often have a longer time on iMV [21], and increased antibiotic consumption [22], resulting in substantial hospital and ICU admission costs, which can vary between 9000 € and 13,000 € per episode [23]. Therefore, prevention is paramount and involves shortening iMV duration, ICU LOS, reducing unnecessary sedation, and upholding rigorous hygiene practices [24].

Despite these concerns, data on VAP incidence and mortality data in Portuguese ICUs remain largely limited. Uncertainty remains about the typical clinical course of afflicted patients, including infection outcome, antimicrobial resistance patterns of commonly involved pathogens, and empiric antimicrobial treatment practices across the country. Thus, this study is the first, to the best of our knowledge, to assess the contemporary VAP epidemiology in Portugal, aiming at improving diagnostic and treatment decisions, especially empiric antimicrobial selection and risk management. Furthermore, this study serves a dual objective by appraising the economic burden of VAP on the national healthcare system, thereby providing a comprehensive understanding of the broader impact of VAP that extends beyond clinical considerations.

## 2. Results

### 2.1. Demographic and Clinical Characteristics

This study included 197 VAP and 197 non-VAP patients from eleven Portuguese hospital facilities. Demographic and clinical characteristics of the patients are shown in Table 1. VAP patients were younger and were more often admitted due to a medical or trauma episode. Their mean SAPS II scores were slightly lower compared to non-VAP patients (50.3 vs. 53.6; *p* = 0.048). The cumulative VAP incidence was 9.2% (95% confidence interval, CI, 8.0–10.5). The median time from ICU admission to VAP diagnosis was 7 [interquartile range, IQR, 5] days. Male patients had a higher risk of developing VAP (OR 1.6, 95% CI 1.34–1.95).

According to the logistic regression model, both gender and cause of admission were independently associated with a higher risk of VAP development (*p* < 0.0001) (Supplementary Table S2).

### 2.2. Healthcare Resource Utilization

Patients with VAP had more than double the LOS, either in ICU or hospital, when compared to non-VAP patients, even after excluding non-survivors. Additionally, VAP patients experienced significantly longer time of iMV (16 [IQR 14] vs. 6 [IQR 6] days, *p* < 0.001) (Table 2). The use of prone positioning (5.1 vs. 0.5%; *p* < 0.001) or tracheostomy (36.5 vs. 14.2%; *p* < 0.001) was also more frequent in the VAP group. All patients with VAP who underwent renal replacement therapy (RRT, *n* = 23) did so after the diagnosis of VAP. The three patients in this cohort who underwent ECMO (extracorporeal membrane oxygenation) support were in the VAP group.

### 2.3. Antimicrobial Treatment

Early-onset VAP was diagnosed in 24.9% of the VAP patients, while late-onset VAP was identified in 75.1%. The rate of microbiological documentation was identical in both groups (85.7% and 81.8%, *p* = 0.525), although the causative microorganisms were significantly different (*p* = 0.035). *S. aureus* was more commonly isolated in early-onset VAP (24.5% vs. 15.5%) while *P. aeruginosa* was more frequently isolated in late-onset VAP (early 12.2%; late 20.9%). *K. pneumoniae* (8.2% vs. 8.8%) was similarly isolated in both settings.

Overall, *P. aeruginosa* and *S. aureus* were the most prevalent isolates (Supplementary Table S1). Although the reported numbers are small, when focusing on hospitals with more than five episodes of either *S. aureus* or *P. aeruginosa*, there seems to be a tendency towards clustering. Among 33 episodes in hospitals 2 and 3, 26 (79%) were caused by *P. aeruginosa*, whereas hospitals 9 and 11 reported 16 cases, of which 12 (75%) were due to *S. aureus*. Hospital 7 reported seven cases, three were due to *P. aeruginosa* and four to *S. aureus*.

Empirical antibiotics were inappropriate in 44.9% and 31.0% of *S. aureus* and *P. aeruginosa* cases, respectively. Demographic and clinical characteristics of appropriate and inappropriate antibiotic therapeutic groups are described in Table 3.

As much as one-third of patients received initial inappropriate empirical antimicrobial treatment, and their 28-day mortality was significantly higher, 34.3% vs. 19.5% (odds ratio 2.16, 95% CI 1.10–4.23, *p* = 0.023). Overall, in 50% of VAP cases, antimicrobial treatment was changed within a median of 3 [IQR 2] days after the diagnosis.

### 2.4. Mortality

The mortality rates among VAP patients at 28, 90, and 365 days were 27.4% (95% CI 21–34), 34.0% (95% CI 27–40), and 40.6% (95% CI 34–48), respectively. Importantly, these mortality rates did not significantly differ from those observed in non-VAP patients (*p *= 0.561, *p *= 0.915, and *p *= 0.918, respectively). Hospital mortality was also similar, 33.5% in the VAP group and 32.5% in the non-VAP group (*p *= 0.574).

Inappropriate antibiotic therapy was associated with 28-day all-cause mortality, 34.3% vs. 19.5% (odds ratio 2.16, 95% CI 1.10–4.23).

However, after 1 year of follow up, in patients with VAP, age, gender, SAPS II, and SOFA scores, but not inappropriate antibiotic therapy (*p* = 0.107 by chi-square test), remain associated with all-cause mortality (Table 4; Supplementary Table S3). Mortality rates in early and late-onset VAP were similar: 38.8% and 41.2%, respectively (*p* > 0.05).

The crude mortality rate according to VAP etiology was as follows: *P. aeruginosa* 44.4%, *S. aureus* 29.4%, and Enterobacterales 43.6%.

## 3. Discussion

To the best of our knowledge, this is the first study to address the epidemiology, mortality rate, and healthcare resource utilization associated with iMV, particularly with VAP, in Portugal. Accordingly, the repercussions on patients’ morbidity and mortality, as well as on the Portuguese healthcare system attributed to a potentially preventable condition, such as VAP, were reported.

In this multicenter study, the prevalence of VAP of 9.2%, and these patients had an all-cause mortality of 24.9% after 28 days, which increased to 40.6% after one year. Notwithstanding, these mortality rates were similar to the control group with iMV > 48 h. These data reflect ICU patients’ case mix in Portugal, along with healthcare resources, which already has been shown to strongly impact VAP prevalence and outcomes [25].

The burden of VAP remains a persistent challenge in critical care settings worldwide. The results herein described align with global trends, where the substantial impact of VAP on increased morbidity and healthcare costs is well documented [12]. This research underscores the considerable significance of VAP as a prevalent nosocomial infection among ICU patients undergoing iMV in Portugal. A better understanding of VAP development risk factors is quite important for anticipating the occurrence of VAP and helping clinical teams implement adequate prevention strategies and control measurements [26].

In our study, patients with or without VAP did not have significant differences in mortality. Nevertheless, patients with VAP had a much longer time on iMV (more than double), as well as a significantly longer ICU and hospital LOS. Consequently, this had a significant impact on patient health conditions and recovery, along with a significant increase in healthcare costs.

Male gender had an increased VAP risk, 1.6 times higher, aligned with findings from previously published data [16]. Most cases of VAP occurred in patients aged 50 years and above, showing that age can facilitate VAP development and is also associated with higher mortality. These findings are in agreement with other studies showing age as a significant risk factor for VAP development and associated mortality [26].

Age may lead to immunosenescence and to a decrease in the ability to clear secretions from the respiratory tract, which may concur with a higher risk of infection, namely VAP. Furthermore, they often experience more severe disease. Patients with more severe disease, independent of age, are often subjected to more invasive procedures, require deeper sedation, and time on iMV, and are more prone to VAP.

Prolonged time on iMV often results from neurotrauma or very severe disease, conditions also known to be associated with VAP. However, the prognosis of these patients is more related to the severity of the underlying condition than to the infection itself.

In our cohort, age and SAPS II score were associated with all-cause mortality after VAP diagnosis. These associations were also found by other groups, with a poor prognosis of VAP in elderly patients and those with a more severe disease [27]. The absence of differences in mortality at 28, 90, and 365 days between VAP and non-VAP patients in our population may be partially explained by differences in underlying critical conditions that may not be captured by SAPS II alone. In fact, others have also observed this lack of association of VAP with mortality, which may also be related, at least in part, to the unspecific diagnostic criteria used. Diagnosis criteria for VAP can influence the diagnosis itself and proper treatment to be started [10].

The high global mortality in all our population may be, in part, due to the paucity of ICU beds available in the Portuguese health system, when compared to other European countries [28], which may lead to the selection of sicker patients. The high SAPS II scores recorded in both groups support this hypothesis.

The all-cause mortality of patients with VAP remains high, ranging from over 30% to 35% at 90 and 180 days, respectively [27]. Further, more severe patients may be exposed to a shorter time on iMV (because of earlier death), paradoxically decreasing the occurrence of VAP, as elegantly demonstrated by Bekaert et al. [29]. In this cohort, death occurred seven days earlier in patients without VAP, again suggesting an occult more severe stage of the disease in the control group, that offset the effect of VAP on mortality in our study.

The association between mortality and VAP has not been consensual, due to the observed heterogeneity in the involved populations. More detailed sub-analysis may be necessary to understand which are the most impactful variables that influence VAP mortality in the different subpopulations. Despite this, high rates suggest a need to analyze and define new strategies to reduce the impact on patients’ outcomes [30]. The number and training of healthcare professionals, healthcare infection prevention programs implementation, hand hygiene procedures, and invasive procedures applied can modulate the mortality of VAP patients, considering the hospital resources and monitoring protocols established [18,31].

The resource consumption associated with VAP cases in the Portuguese cohort was much higher, as previously demonstrated. Other studies reported increased time on iMV, between 7.6 and 11.5 days, and in-hospital LOS between 11.5 and 13.1 days [32,33], leading to an estimated excess cost of approximately 50,000 US dollars per VAP episode [32]. This study mirrors those trends, with VAP patients experiencing higher ICU and hospital LOS and extended iMV periods (16 vs. 6 days). Moreover, complex, and resource-consuming interventions like tracheotomy, prone positioning, or ECMO were significantly more common in VAP patients.

One of the most striking results of our study was the high rate of inappropriate antibiotic therapy. One in three patients with VAP received initial inappropriate empirical antibiotic treatment, and that was associated with increased 28-day mortality (OR 2.16, 95% CI 1.10–4.23). Notwithstanding, inappropriate empirical antibiotic treatment was not independently associated with 1-year all-cause mortality (Table 4). It is also important to note that no relation was found between previous hospital LOS and inappropriate empiric antibiotic treatment.

In our cohort, there was a high rate of microbiological documentation (35.3%), with more than 80% of the patients having a causative agent identified. The most prevalent microorganisms were *P. aeruginosa* and *S. aureus*. Epidemiological studies about VAP agents confirm these agents, together with *K. pneumoniae*, as the main causes of VAP. However, the increasing resistance rates of Enterobacterales, especially of *K. pneumoniae* along with the difficult-to-treat *P. aeruginosa* leads to an increasingly complex empirical antibiotic treatment [20]. This epidemiology of microbiological agents and their resistance profile has a huge impact on the definition of empirical treatments and, consequently, on patients’ outcomes [31]. Changes in microbiology epidemiology and antibiotic resistance should be monitored closely to have appropriate antibiotic treatments, according to these profiles, and to avoid increasing antibiotic resistance and treatment periods.

Avoiding antibiotic therapy whenever possible (to prevent the selection of resistant bacteria) and preventing cross-transmission of resistant bacteria is of paramount importance to decrease this problem. Specific criteria need to be employed to distinguish between colonization and infection, particularly when it affects the decision to prescribe antibiotics that may not be appropriate [34].

Empirical antibiotics were inappropriate in 44.9% of *S. aureus* and 31.0% of *P. aeruginosa* cases. This reinforces the need to have updated data on antimicrobial resistance profiles in ICUs and hospitals, to optimize the empirical treatment according to local epidemiology [35]. Patients receiving appropriate initial treatment had lower crude mortality. The high rate of empirical antibiotic inappropriation may be related to the scarce antimicrobial epidemiological data in the different participating institutions and the absence of adaptative treatment protocols. Notwithstanding, this high rate of inappropriation and the high mortality associated should foster the use of new, more rapid, microbiological tests (e.g., polymerase chain reaction), that were mostly not available at the time of data collection.

Regarding colistin testing, we should remember that although it is sometimes used as a backup drug for extensively resistant organisms, it has significant toxicity [36] and susceptibility testing is fraught with difficulties [37]. Consequently, its use should always be carefully considered, particularly given the recent availability of safer and effective alternatives.

The variations in VAP incidence and outcome across countries and ICU types highlight the intricate interplay of factors influencing its occurrence. In our multivariate model, variables associated with all-cause mortality included age, cause of admission, chronic kidney or liver disease, immunosuppression, and SAPS II score. Previous studies have established similar correlations between age, malnutrition, severe sepsis/septic shock, bacterial infection, bilateral pulmonary infiltrates, and underlying COPD and mortality in VAP patients [29,32].

This study is constrained by its observational and retrospective design, as well as its dependence on ICU discharge notes and medical records, creating a potential for significant bias. Patients were selected based on clinical diagnosis, but no systematic screening was performed. The comparator group was based only on the length of iMV, and selection bias may have been introduced. Moreover, the VAP and non-VAP groups demonstrate heterogeneity in baseline (demographic and clinical) characteristics, which should be considered during data interpretation. Nonetheless, this study offers a comprehensive multicenter and longitudinal perspective, providing pioneer and valuable insights about VAP epidemiology and its impact on Portuguese ICUs.

## 4. Materials and Methods

### 4.1. Study Design

A national, multicenter, retrospective, and longitudinal study was conducted. This study aimed to determine the all-cause mortality rate at 28, 90, and 365 days following ICU admission of patients with or without a clinical diagnosis of VAP. All adult patients admitted to a participating ICU between 1 July 2016 and 31 December 2017, who underwent iMV for at least 48 h, were screened for the presence of VAP, either early onset (occurring within four days) or late onset (five or more days after starting iMV) [38]. The non-VAP group comprised the first consecutive patients ventilated for >48 h who did not develop VAP. The VAP cases were identified based on the patients’ ICU discharge notes or medical records. Only the first VAP episode per patient could be included. Cumulative incidence was computed according to the number of ICU admissions during the study period. Patients with a community- or hospital-acquired pneumonia diagnosis before or within 48 h following iMV initiation, pregnant women, and patients participating in an interventional clinical study during the observation period were excluded.

Sociodemographic and VAP-related clinical data were collected, including baseline comorbidities, short- and long-term mortality, and healthcare resource use. Empirical antimicrobial therapy was assessed, including its appropriateness (according to the isolated microorganisms). Inappropriation was defined as the absence of empirical antimicrobial therapy with at least one active antibiotic against the isolated microorganism. All patients were followed until death or up to 365 days after VAP diagnosis or iMV initiation (among non-VAP patients).

All sites’ Ethical Committees approved this study. Given the non-interventional nature of this study, the data collection based on available information obtained by the participating sites as per routine practice, the data analysis in an anonymized fashion, and the presentation of the results in an aggregate form, informed consent was waived by all participating sites.

### 4.2. Statistical Analysis

All data were summarized using descriptive statistics. For continuous variables, normal distribution tests were applied (Kolmogorov–Smirnoff test), with a 95% level of confidence. Relative risk was calculated to identify risk factors comparing VAP and non-VAP groups (confidence interval (CI) of 95%).

According to data distribution, quantitative variables were compared with the *t*-test for independent samples or the Mann–Whitney non-parametric test. The comparison of qualitative variables was performed with the chi-square test or Fisher’s exact test. A level of confidence of 95% for each test was applied on bivariate models.

The risk of developing VAP was determined by calculating the relative risk, with a 95% CI. Multivariable logistic regression was conducted to explore independent risk factors for VAP development and to explore all-cause mortality-associated factors. Variables with *p* < 0.2 from the bivariate analysis were considered for this examination. A significance level of 0.05 was considered significant and a 95% confidence interval was computed. Data were analyzed by using SPSS Statistics for Windows, Version 23.0 (IBM SPSS Statistics for Windows, Version 23.0. Armonk, NY, USA: IBM Corp.).

## 5. Conclusions

In conclusion, VAP remains prevalent in Portuguese ICU patients and a public health issue with multiple variables to be considered, such as the association with significantly increased use of health resources and costs. Moreover, inappropriate empirical antibiotic therapy was also found to be common and may be associated with potentially worse health outcomes. To the best of our knowledge, this is the largest VAP study published in Portugal and accounts for contemporary data that provide the epidemiology and etiology in Portugal, with further impact on the quality and safety of patient healthcare.

## Figures and Tables

**Table 1:** Demographic and clinical characteristics of VAP and non-VAP patients.

Characteristics		VAP (*n* = 197)	Non-VAP (*n* = 197)	*p*-Value
Age group years, % (*n*)	<50	28.4 (56)	15.2 (30)	0.021 *
	50–69	37.6 (74)	38.0 (75)	
	≥70	34.0 (67)	46.7 (92)	
Gender % (*n*)	Male	79.2 (156)	56.9 (112)	<0.001 *
	Female	20.8 (41)	43.1 (85)	
Comorbidities % (*n*)	COPD	5.0 (10)	8.1 (16)	0.223 *
	Diabetes mellitus	23.9 (47)	23.3 (46)	0.905 *
	Chronic cardiac failure	13.7 (27)	16.2 (32)	0.480 *
	Chronic kidney disease	6.1 (12)	11.7 (23)	0.051 *
	Chronic liver disease	5.6 (11)	4.1 (8)	0.480 *
	Immunosuppression	11.1 (22)	15.2 (30)	0.233 *
ICU admission type % (*n*)	Trauma	24.4 (48)	13.2 (26)	<0.001 *
	Surgical	13.2 (26)	39.6 (78)	
	Medical	37.0 (73)	28.9 (57)	
	Neurocritical	25.4 (50)	18.3 (36)	
SOFA score (max 48 h before VAP diagnosis)	mean (SD)	8.2 (3.4)	8.5 (3.7)	0.392 **
SAPS II	mean (SD)	50.3 (15.9)	53.6 (17.1)	0.048 **

COPD, Chronic Obstructive Pulmonary Disease; ICU, intensive care unit; SAPS, Simplified Acute Physiology Score; SD, Standard Deviation; SOFA, Sequential Organ Failure Assessment; VAP, ventilator-associated pneumonia; * chi-square test; ** Student’s *t* test.

**Table 2:** Healthcare resource utilization.

Characteristics		VAP (*n* = 197)	Non-VAP (*n* = 197)	*p*-Value
Hospital LOS (Days until discharge or death)	Median (Percentile25–Percentile75)	39.0 (22–68)	23.0 (12–47)	<0.001 *
ICU LOS (Days until discharge or death)	Median (Percentile25–Percentile75)	20.0 (13–30)	8 (5–13)	<0.001 *
iMV duration (Days)	Median (Percentile25–Percentile75)	16.0 (11–25)	6.0 (4–10)	<0.001 *
Reintubation in the 60 days after VAP diagnosis, % (*n*)		14.7 (29)	-	-
Prone positioning during ICU admission, % (*n*)		5.1 (10)	0.5 (1)	<0.001 **
Extracorporeal membrane oxygenation during ICU admission (after iMV initiation), % (*n*)		1.5 (3)	0 (0)	-
Renal replacement therapy during ICU admission (after iMV initiation), % (*n*)		11.7 (23)	13.7 (27)	0.544 **

ICU, intensive care unit; iMV, invasive mechanical ventilation; LOS, length of stay; VAP, ventilator-associated pneumonia; * Mann–Whitney non-parametric test; ** chi-square test.

**Table 3:** Demographic and clinical characteristics of “Appropriate Therapy” and “Inappropriate Antibiotic Treatment” VAP patients (*n* = 190).

Characteristics		Appropriate Therapy (*n* = 123)	Inappropriate Therapy (*n* = 67)	*p*-Value
Age group years, % (*n*)	<50	25.4 (31)	34.3 (23)	0.805
	50–69	39.8 (49)	34.3 (23)	
	≥70	35.2 (43)	31.3 (21)	
VAP Onset % (*n*)	Early onset	22.8 (28)	28.4 (19)	0.39
	Late onset	77.2 (95)	71.7 (48)	
Gender % (*n*)	Male	78.9 (97)	80.6 (54)	0.777
	Female	21.1 (26)	19.4 (13)	
Admission % (*n*)	Medical	42.3 (52)	28.4 (19)	0.016
	Neurocritical	26.0 (32)	20.9 (14)	
	Surgical	13.8 (17)	11.9 (8)	
	Trauma	17.9 (22)	38.8 (26)	
Comorbidities % (*n*)	COPD	6.5 (8)	1.5 (1)	0.120
	Diabetes mellitus	20.3 (25)	26.9 (18)	0.303
	Chronic cardiac failure	17.9 (22)	7.5 (5)	0.049
	Chronic kidney disease	6.5 (8)	6 (4)	0.885
	Chronic liver disease	5.7 (7)	3 (2)	0.402
	Immunosuppression	12.2 (15)	10.4 (7)	0.719
SOFA score (max 48 h before VAP diagnosis)	Mean (SD)	8.39 (0.309)	8.80 (0.510)	0.876
SAPS II	Mean (SD)	49.87 (1.489)	53.53 (2.157)	0.225

COPD, Chronic Obstructive Pulmonary Disease;SAPS, Simplified Acute Physiology Score; SD, Standard Deviation; SOFA, Sequential Organ Failure Assessment; VAP, ventilator-associated pneumonia.

**Table 4:** Factors associated with all-cause mortality after VAP diagnosis.

Characteristics		Death by Any Cause after VAP			*p*-Value
		Yes	No	Total	
Age group years, % (*n*)	<50	10	46	56	<0.001 *
	50–69	33	41	74	
	≥70	37	30	67	
Gender % (*n*)	Female	16	25	41	0.05 **
	Male	64	92	156	
Appropriate Antibiotic treatment	Yes	44	79	123	0.107 **
	No	32	35	67	
SAPS II (at ICU admission)	N	80	117	197	0.02 *
	Mean (SD)	53.46 (15.5)	48.14 (16.01)	-	
SOFA score (48 h before VAP)	N	80	117	197	0.05 *
	Mean (SD)	8.54 (3.37)	7.93 (3.34)	-	

ICU, intensive care unit; SAPS, Simplified Acute Physiology Score; SD, Standard Deviation; SOFA, Sequential Organ Failure Assessment; VAP, ventilator-associated pneumonia; * by Student’s *t* test; ** by chi-square tests.

## Data Availability

Data are available upon request from the corresponding author.

## Supplementary Materials

The following supporting information can be downloaded at: https://www.mdpi.com/article/10.3390/antibiotics13040290/s1, Table S1: Isolated microorganisms related to the VAP/antimicrobial susceptibility profile; Table S2: Factors associated with VAP development according to the logistic regression model; Table S3: Factors associated with 1-year all-cause mortality according to the logistic regression model.
