# Supplementary material for: Epidemiology and Burden of Ventilator-Associated Pneumonia among Adult Intensive Care Unit Patients: A Portuguese, Multicenter, Retrospective Study (eVAP-PT Study)
Source: Antibiotics (Basel). 2024 Mar 22;13(4):290. doi: 10.3390/antibiotics13040290 (PMC11047600; doi:10.3390/antibiotics13040290)
Supplement: Supplementary file 1 [file antibiotics-13-00290-s001.zip › antibiotics-2896326-supplementary.pdf]

antibiotics

Epidemiology and Burden of Ventilator-Associated Pneumonia among Adult Intensive Care Unit Patients: A Portuguese, Multicenter, Retrospective Study (eVAP-PT Study)

Paulo Mergulhão [1,†], João Gonçalves Pereira [2,†], Antero Vale Fernandes [3], Andriy Krystopchuk [4], João Ribeiro [5], Daniel Miranda [6], Heloísa Castro [7], Carla Eira [8], Juvenal Morais [9], Cristina Lameirão [10], Sara Gomes [11], Dina Leal [12], Joana Duarte [13], Leonor Pássaro [13], Filipe Froes [14] and Ignacio Martin-Loeches [15,*]

1 Intensive Care Unit, Hospital Lusíadas Porto, 4050-115 Porto, Portugal;paulo.mergulhao.gomes@lusiadas.pt
2 Intensive Care Unit, Hospital de Vila Franca de Xira, 2600-009 Vila Franca de Xira, Portugal; joao.goncalvespereira@hvfx.min-saude.pt
3 Intensive Care Unit, Hospital Garcia de Orta, 2805-267 Almada, Portugal; antero.fernandes@hgo.min-saude.pt
4 Intensive Care Unit, Centro Hospitalar Universitário do Algarve, 8000-386 Faro, Portugal; akrystopchuk@chalgarve.min-saude.pt
5 Intensive Care Unit, Hospital de Santa Maria, Centro Hospitalar Universitário Lisboa Norte, 1649-035, Lisboa, Portugal; jmribeiro@ulssm.min-saude.pt
6 Intensive Care Unit, Centro Hospitalar Vila Nova de Gaia e Espinho, 4434-502 Vila Nova de Gaia, Portugal; daniel.miranda@chvng.min-saude.pt
7 Intensive Care Unit, Hospital de Santo António, Centro Hospitalar Universitário do Porto, 4099-001 Porto, Portugal; u08484@chporto.min-saude.pt
8 Intensive Care Unit, Centro Hospitalar Tondela Viseu, 3504-509 Viseu, Portugal; carla.eira.6452@hstviseu.min-saude.pt
9 Intensive Care Unit, Hospital São Francisco Xavier, Centro Hospitalar Lisboa Ocidental, 1449-005 Lisboa, Portugal; juvenal.jf.morais@azores.gov.pt
10 Intensive Care Unit, Centro Hospitalar Trás-os-Montes e Alto Douro, 5000-508 Vila Real, Portugal; cgomes@chtmad.min-saude.pt
11 Intensive Care Unit, Hospital Prof. Doutor Fernando Fonseca, 2720-276 Amadora, Portugal; sara.r.gomes@hff.min-saude.pt
12 Intensive Care Unit, Hospital de Braga, 4710-243 Braga, Portugal; dina.leal@hb.min-saude.pt
13 Medical Affairs Department, MSD Portugal, 2770-192 Oeiras, Paço de Arcos, Portugal; joana.duarte@merck.com (J.D.); leonor.passaro@ulsetejo.min-saude.pt (L.P.)
14 Intensive Care Unit, Hospital Pulido Valente, Centro Hospitalar Universitário Lisboa Norte, 1769-001 Lisboa, Portugal; filipe.froes@ulssm.min-saude.pt
15 Department of Intensive Care Medicine, Multidisciplinary Intensive Care Research Organization (MICRO), St James' Hospital, Leinster D08NYH1, Dublin, Ireland; imartinl@tcd.ie
* Correspondence: imartinl@tcd.ie
† The authors contributed equally to this work.

Supplementary Materials

Table S1. Isolated microorganisms related to VAP (Antimicrobial susceptibility profile).

Species	Frequency		Antimicrobial Susceptibility		
	%	N		%	N*
Pseudomonas aeruginosa	18.8	42	Ceftazidime	62.2	37
			Piperacillin/Tazobactam	59.5	37
			Imipenem	50	16
			Meropenem	61.5	13
			Gentamicin	80	40
			Amikacin	80	10
			Ciprofloxacin	88.2	34
			Colistin	100	4
Staphylococcus aureus (41% MRSA)	22	49	Amoxicillin Clavulanic	92.3	12
			Oxacillin	59.2	49
			Gentamicin	100	24
			Clindamycin	76.2	21
			Linezolid	100	9

			Cotrimoxazole	100	29
			Vancomycin	100	24
			Ciprofloxacin	76.2	21
			Penicillin	22.2	27
Klebsiella pneumoniae (19% CBR)	9.9	22	Ampicillin	0	16
			Amoxicillin Clavulanic	58.8	17
			Cefuroxime	61.9	21
			Ceftriaxone	66.7	6
			Piperacillin/Tazobactam	27.3	11
			Ertapenem	62.5	8
			Imipenem	66.7	6
			Meropenem	80	10
			Gentamicin	80	20
			Amikacin	100	6
			Cotrimoxazole	58.8	17
			Ciprofloxacin	57.1	14
			Colistin	100	4
Escherichia coli	9.4	21	Ampicillin	46.7	15
			Amoxicillin Clavulanic	42.9	14
			Cefuroxime	66.7	21
			Ceftriaxone	60	5
			Piperacillin/Tazobactam	63.6	11
			Ertapenem	100	7
			Imipenem	100	6
			Meropenem	100	8
			Gentamicin	100	18
			Cotrimoxazole	75	16
			Ciprofloxacin	100	10
Serratia marcesens	7.6	17	Ampicillin	0	9
			Amoxicillin Clavulanic	0	16
			Cefuroxime	0	7
			Ceftriaxone	100	3
			Ceftazidime	71.4	7
			Piperacillin/Tazobactam	83.3	12
			Ertapenem	100	7
			Meropenem	100	7
			Gentamicin	93.3	15
			Cotrimoxazole	90.9	11
Haemophilus influenzae	7.2	16	Ampicillin	46.2	13
			Amoxicillin Clavulanic	92.3	13
			Cefuroxime	100	4
			Ceftriaxone	100	8
			Erythromycin	66.7	3
			Cotrimoxazole	100	15
			Levofloxacin	100	3
Enterobacter aerogenes	3.6	8	Ampicillin	0	6
			Amoxicillin Clavulanic	0	7
			Cefuroxime	60	5
			Cefotaxime	60	5
			Piperacillin/Tazobactam	40	5
			Ertapenem	100	3
			Imipenem	100	4
			Meropenem	100	4
			Gentamicin	87.5	8
			Cotrimoxazole	83.3	6
			Ciprofloxacin	100	7

Other	21.6	48	-	-	-

CBR, Carbapenem Resistant; MRSA, Methicillin-resistant *Staphylococcus aureus*; *Number of species of each microorganism that have susceptibility reported to each antibiotic. Percentage of susceptibility refers only to these reported samples.

Table S2. Factors associated with VAP development according to the logistic regression model.

Regression Model	Model 1			Model 2		Model 3	
	**Beta**	**CI 95%**	***p-value***	**Beta**	***p-value***	**Beta**	***p-value***
Intercept	0.792			0.465		0.767	
Gender	0.395	[0.246; 0.634]	<0.0001	0.391	<0.0001	0.385	<0.001
Admission type	0.223	[0.114; 0.438]	<0.0001	0.228	<0.0001	0.221	<0.001
Chronic kidney disease	1.379	[0.624; 3.046]	0.427	1.36	0.446		
Liver disease	0.711	[0.26; 1.940]	0.505				

CI: confidence interval.

Table S3. Factors associated with 1-year all-cause mortality according to the logistic regression model.

Regression Model	Model 1		Model 2		Model 3		Model 4		Model 5	
	**Beta**	***p-value***	**Beta**	***p-value***	**Beta**	***p-value***	**Beta**	***p-value***	**Beta**	***p-value***
Intercept	−3,329		−3,261		−3,048		−2,665		−1,654	
Age group	0.104	<0.001	0.362	<0.001	0.354	0,001	0.38	<0.001	0.322	<0.001
Gender	0.222	0.576	0.236	0.551						
Appropriate antibiotic treatment	0.633	0.057	0.627	0.058	0.627	0.058	0.633	0.061		
SOFA score	0.019	0.711								
SAPSII Score	0.008	0.468	0.01	0.326	0.01	0.322				

SAPS, Simplified Acute Physiology Score; SOFA, Sequential Organ Failure Assessment.
